# Educational inequalities in young-adult mortality between the 1990s and the 2000s: regional differences in Belgium

**DOI:** 10.1186/s13690-014-0059-3

**Published:** 2015-03-16

**Authors:** Hannelore De Grande, Hadewijch Vandenheede, Patrick Deboosere

**Affiliations:** Department of Sociology – Interface Demography, Vrije Universiteit Brussel, Pleinlaan 5, 1050 Brussels (Elsene), Belgium

**Keywords:** Educational inequalities, Absolute and relative differences, Adolescent & young-adult mortality, Regional differences

## Abstract

**Background:**

This study addresses educational inequalities in young-adult mortality between the 1990s and the 2000s by comparing trends in the three different regions in Belgium stratified by sex. Social inequalities in mortality are of major concern to public health but are rarely studied at young ages. Substantial health differences have been found between the Flemish (FR) and Walloon region (WR) concerning (healthy) life expectancy and avoidable mortality, but little is known about regional differentials in young-adult mortality, and comparisons with the Brussels-Capital Region (BCR) have thus far never been made.

**Methods:**

Data are derived from record linkage between the Belgian censuses of 1991 and 2001 and register data on death and emigration for the periods 01/03/1991-01/03/1999 and 01/10/2001-01/10/2009. Analyses are restricted to young adults aged 25 to 34 years at the moment of each of the censuses. Absolute (directly standardized mortality rates (ASMRs)) and relative (mortality rate ratio using Poisson regression) measures were calculated.

**Results:**

There is a significant drop in young-adult mortality between the 1990s and the 2000s in all regions and both sexes, with the strongest decline in the BCR (e.g. ASMR of men declined from 165.6 [151.1-180.1] per 100,000 person years to 73.8 [88.3-98.3]). The mortality rates remain highest in the WR in the 2000s Between the 1990s and the 2000s, a remarkable change in the educational distribution occurred as well, with much lower proportions of primary educated in all regions in the 2000s in favour of higher proportions in all other educational levels, especially in higher education. All educational groups show lower mortality over time, except for lower educated men in the FR.

**Conclusions:**

There is a positive evolution towards lower mortality among the young-adult Belgian population. The WR trails behind in this evolution, which calls for tailored preventive actions. Educational inequalities are marked in all regions and time periods. A more general discussion is needed on the responsibility of society in rendering support and capability to enhance the state of well-being of those not able to achieve a high social position.

**Electronic supplementary material:**

The online version of this article (doi:10.1186/s13690-014-0059-3) contains supplementary material, which is available to authorized users.

## Background

Mortality declined steadily in the last decades in most western countries [1-3]. Despite this generally positive evolution, relative inequalities in morbidity and mortality have persisted or widened over time [4-6]. This trend is less obvious in mortality among the young (15- to 34-year olds), with all-cause mortality declines sometimes masking increases in avoidable cause-specific mortality such as suicides, homicides and drug-related deaths [7-10]. Other research however found strong mortality declines over time in all-cause [11] and cause-specific mortality [10,12], following the general trend at older ages.

Education is the most commonly used socioeconomic position (SEP) indicator when studying inequalities in young adulthood [13]. The obtained educational level is strongly determined by parental characteristics such as household income and fathers’ occupation. For this paper the age group of 25–34 is selected because most people in Belgium have completed their formal education by the age of 25. Education is then the most stable indicator for SEP, whereas other measures such as income or occupation are more prone to change in this life stage. The link between education and health/mortality is firmly established e.g. [6,14-16]. From young adulthood onwards, social inequalities increase following a social gradient that continues well into old age [17,18]. Education not only influences health outcomes through increased knowledge and insights but also indirectly through jobs with more benefits, a higher income and through a greater sense of control and social support [16,19,20]. Monitoring educational inequalities in mortality over time provides insight into the interplay of compositional changes in educational groups and inequalities in mortality between educational levels.

Regional differences in absolute and relative mortality inequalities have been observed in several European countries [21]. In Belgium, substantial regional differences have been found in avoidable mortality [22,23] and (healthy) life expectancy [24,25]. Belgium consists of three regions: the Flemish Region (FR – North), Walloon Region (WR – south) and Brussels-Capital Region (BCR – centre). The Walloon Region (WR) does not only have a worse health status than the Flemish Region (FR); inequalities are also larger both in life expectancy and disability free life expectancy [24-26]. The worse economic situation in the WR is put forward as a main contributor to the persisting regional differences [25]. Yet, these studies did not include the BCR and did not have a specific focus on young adults. The BCR is often not included in these comparisons, because of the small population size in research using sampling designs [27]. This restriction does not hold for this paper, as exhaustive population data are used. Research in the BCR using these data found lower mortality rates over time among 15-to-34-year olds and persisting educational inequalities, especially among men [12], but did not compare the different regions. Leaving the BCR out of regional comparisons only allows for a partial insight into regional mortality inequalities, in particular because its metropolitan character entails higher income inequalities [28]. Mechanisms behind social inequalities in Belgium cannot be fully understood without accounting for this region as well.

This study addresses the evolution in educational inequalities in mortality among young adults between the 1990s and the 2000s. This study adds to the literature through its focus on young-adult mortality, on trends over time and through its inclusion of the BCR and thus comparison of the three Belgian regions. We respectively examine regional differences and trends in 1) all-cause mortality in young adults, 2) educational inequalities in young-adult mortality and 3) if the observed regional differences in educational inequalities are due to differences in the population composition between the regions in terms of nationality of origin and employment. These research questions will be dealt with stratified by gender.

## Methods

### Data

Data are derived from two Belgian censuses linked to death and emigration records of the national registry. These provide exhaustive information on the official population living in Belgium at the time of each census collection (01/03/1991 and 01/10/2001). Follow-up is possible due to linkage with national register data on deaths and emigrations for the respective periods 1991–1999 and 2001–2009. The cohort is semi-closed; no new entries (either by immigration or birth) are taken into account.

### Study population

We restrict the analysis to young adults aged 25 to 34 at baseline. We observe two periods, 1991–1999 and 2001–2009, further broken down in two periods each of which contains four years of follow-up time: 01/03/1991-01/03/1995 (P1), 01/03/1995-01/03/1999 (P2), 01/10/2001-01/10/2005 (P3) and 01/10/2005-01/10/2009 (P4). At each new interval, the age range of 25 to 34 years is recalculated to ascertain comparability between periods. For the exposure time calculation, we use age at entry for P1 and P3, and the recalculated age at the beginning of P2 and P4.

### Variables

#### Own educational level

Four categories are used, following the ISCED-classification: 1) no/primary, 2) lower secondary education, 3) higher secondary education, and 4) higher education. Missing values on education are included as separate categories in the analyses. We observe a shift towards higher educational levels over time, in all regions (Table 1). For example, while 20.7% of the Flemish women had a degree of primary education in 1991, this is only 3.8% in 2001. Furthermore, in 2001 there is a higher share of both low and higher educated young persons in the BCR in both sexes compared to the other regions, a trend that is still apparent in recent observations [28].

**Table 1:** **Distribution of population characteristics among young adults (25–34 yrs) in Belgium according to sex, period and region (in column %)**

		**MEN**						**WOMEN**					
		**1991**			**2001**			**1991**			**2001**		
		**FR**	**BCR**	**WR**	**FR**	**BCR**	**WR**	**FR**	**BCR**	**WR**	**FR**	**BCR**	**WR**
**Educational level**													
Primary		17.36	21.79	21.41	4.03	8.48	5.94	20.65	22.98	21.42	3.75	8.86	4.87
Lower sec		21.57	18.83	18.83	15.34	17.60	22.65	15.03	15.45	22.42	11.33	15.04	18.26
Higher sec		35.33	24.40	32.03	45.18	26.94	40.06	35.61	23.71	30.65	41.05	24.31	35.80
Higher		25.74	34.98	20.95	35.45	46.98	31.34	28.72	37.86	25.51	43.86	51.78	41.07
	Missing	(5.17)	(21.91)	(6.71)	(5.07)	(19.35)	(8.36)	(4.56)	(19.53)	(5.26)	(3.80)	(16.99)	(6.08)
**Nationality of origin**													
Belgian		92.68	58.86	78.89	93.38	60.18	80.01	88.49	42.82	77.17	88.15	42.68	77.58
European/Western		4.29	20.11	16.09	3.91	20.45	15.72	5.26	22.24	15.75	5.20	24.58	15.37
Maghrebin/Turkish		1.66	12.88	2.51	1.58	12.14	2.03	3.95	23.52	4.18	3.39	19.59	3.41
Other		1.37	8.15	2.51	1.13	7.23	2.23	2.30	11.42	2.91	3.27	13.14	3.64
**Employment situation**													
Self-employed		13394	14.44	13.08	10.50	8.70	8.09	8.57	7.63	7.63	6.32	4.73	4.42
Employee		35.46	38.28	31.76	42.59	44.21	39.52	45.81	48.52	42.43	55.86	47.67	46.99
(Un) Skilled worker		42.39	25.25	38.57	27.75	14.47	25.07	17.95	9.77	10.70	10.18	4.99	6.30
Job seeker		3.72	11.40	9.69	4.48	16.56	12.09	12.42	15.29	20.40	7.20	19.21	19.43
Not working, not seeking a job		0.82	1.31	1.75	1.80	2.76	2.39	9.39	10.23	13.16	7.74	10.65	10.15
Working, job unknown		1.72	3.32	2.10	9.08	7.93	9.58	2.96	3.23	2.45	7.90	7.29	7.89
Other		1.95	6.01	3.06	3.80	5.37	3.26	3.16	5.54	3.23	4.80	5.46	4.82
	Missing	(3.07)	(18.47)	(5.85)	(3.73)	(17.36)	(6.78)	(2.96)	(15.64)	(4.55	(3.19)	(15.99)	(5.71)

Data: census 1991, 2001 linked to national register, own calculations.

% are calculated exclusive of missings; between brackets: % of missings per indicator compared to the total.

The composition of the young population not only differs regionally in terms of educational level, but also in terms of other factors. The BCR attracts migrants from all over the world and thus consists of a large population of non-Belgian origin, especially in the youngest age groups [28]. The influx of migration in 2001 made differences with 1991 even more substantial (Table 1). Some nationality groups have lower or higher mortality risks compared to the native Belgian population [29,30], which might impact a regional comparison. Nationality of origin is included in four categories in our analyses: 1) Belgian, 2) European/Western (including all EU-15 countries, USA, Canada, Japan, Australia & New Zealand), 3) Maghrebin (all North-African countries except Egypt)/Turkish, and 4) other.

As inequalities in mortality exist between the employed versus the non-employed [16] and the composition of employed/non-employed differs between the regions and increases over time, employment situation is another important factors that needs to be adjusted for. It is operationalized as a combination of information on the type of occupation and being employed or not. It contains seven categories: 1) self-employed, 2) employee, 3) (un) skilled worker, 4) job seeker, 5) not working and not seeking a job, 6) working but unknown sort of job and 7) other (including students, <1% of the total population). There is a shift towards more employees and less (skilled) workers in 2001 compared to 1991. The percentage of (skilled) workers is a lot lower in the BCR than in the other regions for both men and women.

### Analysis

Both relative and absolute inequalities in mortality are calculated. We computed *age-standardised all-cause mortality rates* (ASMRs), directly standardised to the European population of 2013 [31]. Absolute mortality decline is calculated between each period and between the first (1991–1995) and the last period (2005–2009). Relative mortality decline is calculated by dividing the absolute mortality decline of the latter period by the ASMR of the former period. It is calculated between each period and between 1991–1995 and 2005–2009. Furthermore, *mortality rate ratios* (MRRs) are calculated using Poisson regression. In these models, nationality of origin and employment situation are used as controls to test the robustness of the educational inequalities. The Poisson regressions are carried out with STATA MP 13.1 and stratified by region and period. To test if relative inequalities significantly changed over time, the 1991 and 2001 census were integrated to include the interaction between period and educational level (see Table 2). The terminology ‘1990s’ and ‘2000s’ is used to designate the periods 1991–1999 and 2001–2009. If we refer to a specific sub period (e.g. 1991–1995), the period is specified in the text. Each model is also controlled by age and presented separately for men and women.

## Results

### General mortality changes over time

**Figure 1:**
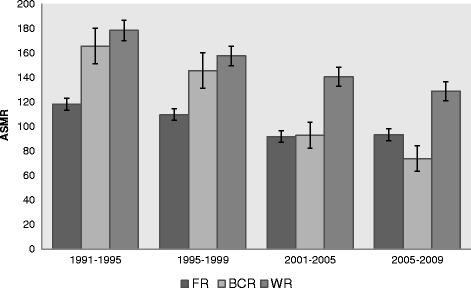
**Age-standardized mortality rates (ASMRs) with confidence intervals per 100,000 person years in young-adult MEN (25-34 yrs) from 1991 until 2009 in the three Belgian regions.** FR= Flemish Region, BCR=Brussels-Capital Region, WR= Walloon Region.

**Figure 2:**
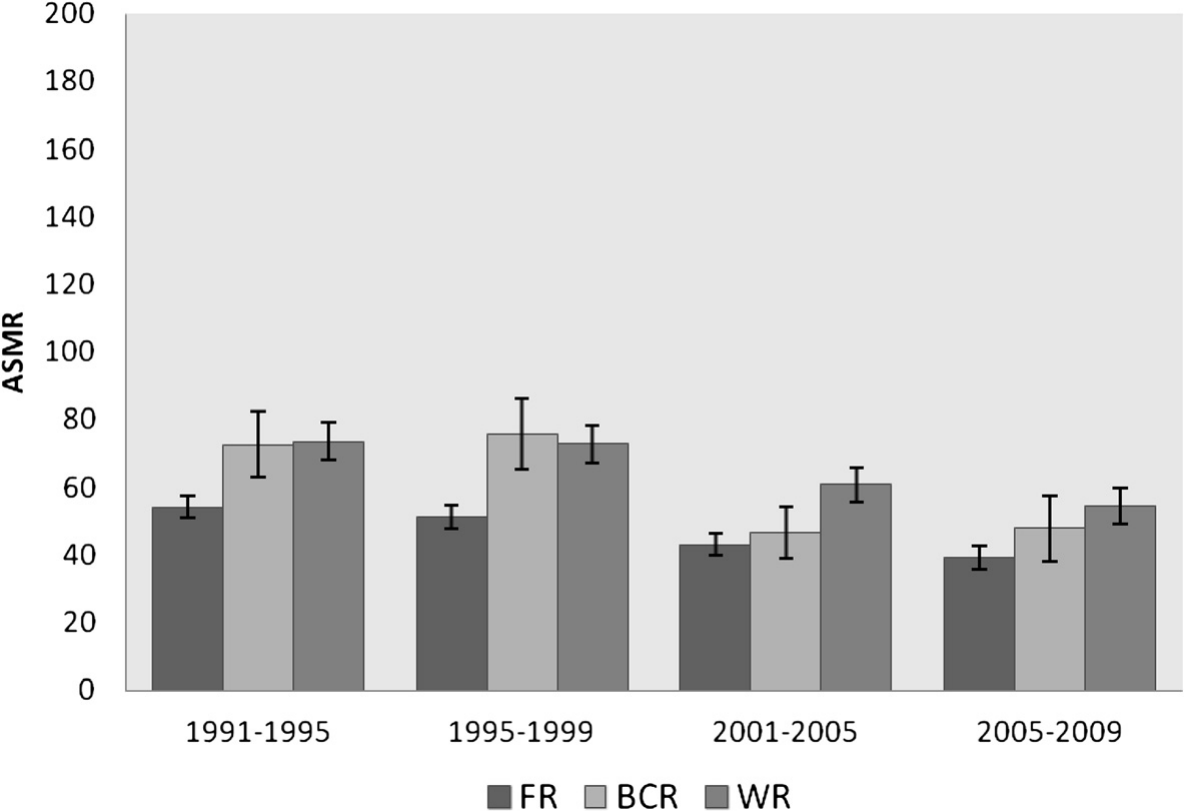
**Age-standardized mortality rates (ASMRs) with confidence intervals per 100,000 person years in young-adult WOMEN (25-34 yrs) from 1991 until 2009 in the three Belgian regions.** FR= Flemish Region, BCR=Brussels-Capital Region, WR= Walloon Region.

To answer the first research question concerning regional differences in all-cause mortality over time, we make use of Figure 1 (men) and Figure 2 (women). Young-adult mortality decreased considerably over time, in all regions, and in both men and women.

In 1991–1995 male mortality rates are clearly smallest in the FR (ASMR_FR-91_ = 118.3 [113.4-123.2]) compared to the other regions, having similar mortality rates (ASMR_BCR-91_ = 165.6 [95% CI 151.1-180.1]; ASMR_WR-91_ = 178.3 [170.0-186.6]). This pattern changes over time, as the mortality decline was most pronounced in men in the BCR. In 2001–2005, mortality rates become similar between the FR and the BCR, and in 2005–2009 mortality is even significantly lower in the BCR than in the FR (ASMR_BCR-05_ = 73.8 [63.8-84.3]; ASMR_FR-05_ = 93.3 [88.3-98.3]). The decline is smallest in the FR, with a relative mortality decline of 21% over the total observation period (1991–2009) compared to 28% in the WR and 55% in the BCR (not in Figure). The WR still shows the highest mortality rates at the end of the observation period (ASMR_WR-05_ = 128.8 [121.0-136.6]).

The mortality pattern among women is similar to that of men, although the decline over time in the BCR is more modest (Figure 2). Relative mortality declines range from 26% in the WR and 27% in the FR to 34% in the BCR (not in Figure). Contrary to the regional mortality pattern among men, young-adult women living in the FR still have the lowest mortality in 2005–2009 (ASMR_FR-05_ = 39.2 [36.0-42.4]).

### Educational mortality pattern over time

Our second research question addresses the regional differences in educational inequalities in all-cause mortality over time. Table 3 shows educational mortality differences over time for men, with person years, mortality rates and number of deaths for each category, period and region. Additional file 1: Table S1 shows the related absolute and relative mortality differences over time. In the FR, mortality declined most among the higher educated (ASMR_FR-05-higher_ = 42.5 [36.8-48.1] compared to ASMR_FR-91-higher_ = 64.2 [56.9-71.6]) (Additional file 1: Table S1). There is a relative mortality increase of 18% among the primary educated and 11% among the lower secondary educated in 2005–2009 compared to 2001–2005 (Table 4). As a result of this, overall mortality decline is smaller in the FR and both absolute and relative inequalities have increased over time. In the BCR, we observe substantial mortality declines in almost all educational levels (especially between 1995 and 2001: ranging from a 26% decrease among the lower secondary educated to 41% among the higher educated – Additional file 1: Table S1). In 2005–2009, inequalities increased again because of an increase in mortality among the primary educated while there are further decreases in the other educational categories. In the WR, there is a strong decrease in educational inequalities in 2001–2005, followed by a small increase in inequalities in 2005–2009.

**Table 2:** **All-cause mortality rate ratios (MRR): interaction terms between educational level and period by region and sex (reference category = higher education)**

		**FR**		**BCR**		**WR**	
		**MRR**	**sign.**	**MRR**	**sign.**	**MRR**	**sign.**
MEN	Higher sec 91	1.69		1.62		1.54	
	Higher sec 01	2.09	*	1.96		2.17	**
	Lower sec 91	1.95		2.04		1.91	
	Lower sec 01	3.01	***	2.53		2.93	**
	Primary 91	2.83		2.28		2.87	
	Primary 01	3.97	*	3.10		3.67	
WOMEN	Higher sec 91	1.28		1.93		1.06	
	Higher sec 01	1.50		1.18		1.53	*
	Lower sec 91	1.56		2.20		1.30	
	Lower sec 01	2.17	*	0.99	*	1.73	
	Primary 91	1.87		2.37		1.86	
	Primary 01	3.67	***	2.35		2.68	

Combined dataset of census 1991 and 2001, own calculations.

FR = Flemish Region, BCR = Brussels-Capital Region, WR = Walloon Region.

Sign. = Statistical significant difference between period 1991–1995 and 2001–2005.

*: p < 0.050; **: p < 0.010; ***: p < 0.001.

**Table 3:** **All-cause age-standardised mortality rates (ASMRs) and confidence intervals (CI) per 100,000 person years (PY) among young-adult MEN (25–34 years) in Belgium for 4 periods of follow-up**

		**1991-1995**				**1995-1999**				**2001-2005**				**2005-2009**			
		**PY**	**ASMR**	**95% CI**	**d**	**PY**	**ASMR**	**95% CI**	**d**	**PY**	**ASMR**	**95% CI**	**d**	**PY**	**ASMR**	**95% CI**	**d**
FR	Higher	462,547	64.2	[56.9-71.6]	294	488,927	60.1	[56.2-66.9]	293	538.763	44.3	[38.6-49.91]	238	517.494	42.5	[36.8-48.1]	218
	Higher sec	639,489	108.6	[100.4-116.7]	686	679,895	105.3	[97.5-113.0]	716	690,424	92.4	[85.2-99.5]	637	647,849	100.1	[92.4-107.8]	648
	Lower sec	389,240	124.3	[113.3-135.4]	484	328,413	127.2	[114.9-139.5]	420	234,054	136.0	[120.8-151.3]	314	185,247	150.6	[132.9-168.3]	279
	Primary	312,359	180.8	[165.9-195.8]	567	227,664	178.3	[160.6-196.1]	403	61,154	178.5	[144.3-212.7]	108	44,120	210.6	[167.7-253.5]	93
	Missing	92,453	219.6	[189.2-250.0]	202	85,805	180.6	[152.2-209.1]	155	73,965	227.0	[192.7-261.3]	168	59,172	197.6	[161.8-233.4]	117
	Total	1,896,088	118.3	[113.4-123.2]	2,233	1,810,704	109.7	[104.9-114.5]	1,987	1,598.360	91.7	[87.0-96.3]	1,465	1,453,882	93.3	[88.3-98.3]	1,355
BCR	Higher	83,373	99.2	[77.3-121.2]	79	81,858	73.2	[54.5-91.9]	60	121,849	43.4	[31.7-55.1]	53	107,015	34.6	[23.4-45.7]	37
	Higher sec	59,620	156.8	[124.2-189.4]	91	59,416	133.0	[104.3-163.7]	79	71,606	86.1	[64.5-107.7]	61	67,354	73.8	[53.2-94.3]	50
	Lower sec	45,681	194.6	[154.2-235.0]	89	37,707	149.9	[111.0-188.8]	57	46,869	111.2	[81.0-141.5]	52	37,048	104.5	[71.7-137.3]	39
	Primary	52,911	220.1	[180.0-260.1]	116	41,532	223.0	[177.4-268.6]	92	22,469	133.1	[85.8-180.4]	31	14.221	159.6	[90.6-228.5]	22
	Missing	63,318	200.0	[165.1-234.9]	127	48,040	209.3	[168.4-250.0]	101	56,121	176.4	[141.7-211.1]	99	35,912	123.9	[87.7-160.1]	45
	Total	304,903	165.6	[151.1-180.1]	502	268,553	145.4	[131.0-159.9]	389	318,915	92.9	[82.4-103.5]	296	261,550	73.8	[63.4-84.3]	193
WR	Higher	193,715	93.3	[79.7-107.0]	180	205,664	60.9	[50.2-71.6]	125	256,107	57.3	[48.0-66.6]	147	252,537	63.4	[53.6-73.2]	160
	Higher sec	299,972	143.	[129.5-156.8]	428	313,941	143.0	[129.7-156.2]	449	329,706	124.8	[112.7-136.8]	411	319,531	116.2	[104.3-128.0]	371
	Lower sec	239,146	178.2	[161.3-195.1]	426	207,759	168.3	[150.6-186.1]	347	186,321	170.3	[151.5-189.1]	316	150,432	190.0	[168.0-211.9]	289
	Primary	199,386	268.8	[246.0-291.6]	537	155,559	268.3	[242.6-294.0]	425	48,784	212.8	[171.5-254.1]	104	35,120	246.0	[193.7-298.3]	87
	Missing	62,989	320.4	[275.8-364.9]	200	58,602	234.0	[194.9-273.2]	137	70,545	387.6	[341.8-433.4]	274	57,422	251.7	[210.8-292.7]	145
	Total	995,208	178.3	[170.0-186.6]	1,771	941,525	157.5	[149.5-165.5]	1,483	891,461	140.5	[132.7-148.2]	1,252	815,041	128.8	[121.0-136.6]	1,052

Data: census 1991, 2001 linked to national register, own calculations.

FR = Flemish Region, BCR = Brussels-Capital Region, WR = Walloon Region.

d = number of deaths.

The educational pattern of women in the FR (Table 4) is comparable to that of men. While the highest educated groups have lower mortality rates in the 2000s, there is an increase in mortality in the lowest educated (e.g. ASMR_FR-91-primary_ = 70.3 [61.8-78.9]), ASMR_FR-01-primary_ = 104.4 [77.5-131.3]). Mortality among the highest educated decreased with 34%, leading to larger inequalities over time (Additional file 2: Table S2). In the BCR, there is a clear split between those with primary education and the other educational groups in 2001–2005. There is only a small mortality decline in the primary educated (8%), while the decline in other groups ranges from 36 to 53%. In 2005–2009, a further mortality decline is only observed in the highest educated women, leading to pronounced differences between the highest educated and the other educational levels. In the WR, trends in educational inequalities are more substantial over time, with increases among the lowest educated (59%) and large decreases over time among the highest educated (44%).

As already detailed in Table 1, a remarkable change in the educational distribution occurred in all regions and in both men and women. Primary educated young adults become a small group in the 2000s (e.g. Additional file 1: Table S1: PY in the primary educated men of the WR in 1991: 199,386 compared to 48,784 in 2001). The overall mortality decline is thus not only the result of a decrease in mortality in most educational groups, but is also due to a shrinking low-educated group with high mortality risks compared to the higher educated. The latter group expanded most over time (e.g. in the BCR, PY in women increased from 94,292 in 1991 to 136,211 in 2001).

### Robustness of regional differences in educational inequalities in mortality after controlling for employment situation and nationality of origin

To answer our third research question, age-adjusted mortality rate ratios (MRRs) are presented in Table 5 (men) and Table 6 (women). Model 1 is adjusted for age, model 2 is controlled for age and employment situation and model 3 for age, employment situation and nationality of origin. Trends are presented by comparing the first period after each census. Analyses for the other periods are not presented because we lack updated information on the employment situation in 1995 and 2005. A full table with this information is available on request.

**Table 4:** **All-cause age-standardised mortality rates (ASMRs) and confidence intervals (CI) per 100,000 person years (PY) among young-adult WOMEN (25–34 years) in Belgium for 4 periods of follow-up**

		**1991-1995**				**1995-1999**				**2001-2005**				**2005-2009**			
		**PY**	**ASMR**	**95% CI**	**d**	**PY**	**ASMR**	**95% CI**	**d**	**PY**	**ASMR**	**95% CI**	**d**	**PY**	**ASMR**	**95% CI**	**d**
FR	Higher	496,695	38.0	[32.5-43.6]	183	561,129	38.8	[33.6-44.0]	214	657,477	27.7	[23.6-31.7]	181	664,075	25.1	[21.3-29.0]	166
	Higher sec	619,162	48.4	[42.8-53.9]	295	649,760	46.1	[408.8-51.3]	297	617,636	41.8	[36.7-46.9]	260	560,428	40.8	[35.5-46.0]	229
	Lower sec	260,170	59.7	[50.4-69.1]	156	211,952	48.5	[39.0-58.1]	102	170,246	61.5	[49.6-73.4]	108	121,249	65.1	[508-79.4]	80
	Primary	358,004	70.3	[61.8-78.9]	264	245,618	88.6	[76.4-100.8]	219	56,266	104.4	[77.5-131.3]	60	39,302	80.0	[51.1-108.9]	30
	Missing	79,179	94.4	[72.8-115.9]	74	74,732	84.2	[63.4-105.0]	63	53,352	115.9	[81.1-144.7]	62	45,163	121.9	[89.7-154.1]	55
	Total	1,813,210	54.2	[50.8-57.6]	972	1,743,191	51.2	[47.9-54.6]	895	1,554,977	42.9	[39.7-46.2]	671	1,430,217	39.2	[36.0-42.4]	560
BCR	Higher	94,292	36.8	[24.2-49.3]	34	93,305	57.0	[41.3-72.7]	52	136,211	36.4	[26.1-46.6]	49	122,711	25.6	[16.6-34.7]	31
	Higher sec	60,133	73.0	[50.8-95.2]	42	57,706	83.7	[59.7-107.8]	47	65,268	42.6	[26.8-58.4]	28	62,336	58.0	[38.6-77.4]	35
	Lower sec	38,751	82.5	[53.9-111.0]	32	29,772	77.8	[46.6-108.9]	24	40,575	36.2	[17.9-54.5]	15	31,780	62.9	[35.3-90.6]	20
	Primary	57,969	89.6	[65.2-114.0]	52	43,490	93.2	[65.2-121.2]	43	23,764	85.9	[49.0-122.8]	21	16,452	107.9	[56.0-159.9]	17
	Missing	57,142	108.2	[81.2-135.8]	61	44,511	77.9	[52.1-103.7]	35	47,725	66.1	[42.8-89.4]	31	32,234	70.8	[41.9-99.8]	23
	Total	308,287	72.7	[63.1-82.4]	221	268,784	75.8	[65.3-86.6]	201	313,543	46.5	[38.9-54.1]	144	265,513	47.8	[39.4-56.1]	126
WR	Higher	233,950	54.4	[44.8-64.0]	125	258,460	50.0	[41.3-58.7]	127	338,390	37.1	[30.6-43.7]	124	347,692	30.3	[24.5-36.1]	105
	Higher sec	283,532	57.0	[48.1-65.9]	160	304,334	53.4	[45.1-61.7]	160	296,338	57.0	[48.4-65.6]	169	279,680	54.3	[45.7-62.9]	92
	Lower sec	206,043	70.8	[59.4-82.3]	147	170.027	84.0	[70.4-97.5]	149	151,268	65.5	[52.6-78.5]	100	111.813	80.0	[63.3-96.6]	92
	Primary	197,123	101.3	[87.3-115.2]	204	141,689	116.5	[98.4-134.6]	166	40,175	102.6]	[70.9-134.2]	42	26,517	161.5	[113.0-209.9]	45
	Missing	48,102	154.6	[119.0-190.3]	73	48,236	149.4	[114.9-183.9]	72	49,447	198.3	[159.0237.5]	98	41,856	118.8	[85.8-151.7]	50
	Total	968,750	73.6	[68.2-79.0]	709	922,746	72.9	[67.4-78.4]	674	875,616	60.8	[55.7-66.0]	533	807,557	54.6	[49.5-59.7]	384

Data: census 1991, 2001 linked to national register, own calculations.

FR = Flemish Region, BCR = Brussels-Capital Region, WR = Walloon Region.

d = number of deaths.

**Table 5:** **All-cause mortality rate ratios and confidence intervals among young-adult MEN in Belgium by region for 1991–1995 and 2001–2005 (reference category = higher education)**

		**91-95**			**01-05**		
		**Model 1**	**Model 2**	**Model 3**	**Model 1**	**Model 2**	**Model 3**
FR	Higher sec	1.69***	1.71***	1.70***	2.09***	1.77***	1.77***
		[1.48,1.94]	[1.48,1.97]	[1.47,1.96]	[1.80,2.43]	[1.50,2.08]	[1.51,2.09]
	Lower sec	1.94***	1.86***	1.87***	3.04***	2.28***	2.32***
		[1.68,2.24]	[1.59,2.19]	[1.59,2.19]	[2.57,3.60]	[1.89,2.75]	[1.92,2.80]
	Primary	2.81***	2.23***	2.27***	4.01***	2.31***	2.48***
		[2.44,3.24]	[1.91,2.61]	[1.94,2.66]	[3.19,5.03]	[1.80,2.95]	[1.93,3.19]
BCR	Higher sec	1.63**	1.68**	1.60**	1.96***	1.68*	1.72**
		[1.20,2.20]	[1.22,2.30]	[1.17,2.20]	[1.36,2.84]	[1.14,2.49]	[1.17,2.55]
	Lower sec	2.04***	1.89***	1.96***	2.53***	1.93**	2.05***
		[1.51,2.76]	[1.36,2.62]	[1.41,2.72]	[1.73,3.71]	[1.27,2.93]	[1.35,3.12]
	Primary	2.27***	1.74***	1.84***	3.11***	2.08**	2.34***
		[1.71,3.03]	[1.26,2.40]	[1.33,2.54]	[2.00,4.85]	[1.29,3.38]	[1.44,3.82]
WR	Higher sec	1.54***	1.62***	1.59***	2.17***	1.90***	1.88***
		[1.30,1.83]	[1.35,1.94]	[1.32,1.90]	[1.80,2.62]	[1.55,2.32]	[1.54,2.29]
	Lower sec	1.91***	1.87***	1.86***	2.94***	2.27***	2.25***
		[1.60,2.27]	[1.55,2.26]	[1.54,2.24]	[2.42,3.58]	[1.83,2.81]	[1.81,2.78]
	Primary	2.86***	2.22***	2.21***	3.69***	2.27***	2.29***
		[2.42,3.39]	[1.84,2.68]	[1.83,2.67]	[2.87,4.75]	[1.73,2.99]	[1.74,3.02]

Data: census 1991, 2001 linked to national register, own calculations.

***: p < 0.001; **: p < 0.010; *: p < 0.050.

FR = Flemish Region, BCR = Brussels-Capital Region, WR = Walloon Region.

Model 1: Controlled by age/Model 2: Controlled by age and employment situation/Model 3: Controlled by age, employment situation and nationality of origin.

Table 5 shows that educational inequality in male mortality is increasing in all regions between 1991–1995 and 2001–2005, especially in primary and lower secondary educated men (e.g. MRR_FR-91-primary-model1_ = 2.81 [2.44-3.24] compared to MRR_WR-01-primary-model1_ = 4.01 [3.19-5.03]). The difference between the periods is statistically significant for the WR and FR (Table 2). In the BCR, MRRs are also higher, but the differences between the periods are less pronounced and not significant.

**Table 6:** **All-cause mortality rate ratios and confidence intervals among young-adult WOMEN in Belgium by region for 1991–1995 and 2001–2005 (reference category = higher education)**

		**91-95**			**01-05**		
		**Model 1**	**Model 2**	**Model 3**	**Model 1**	**Model 2**	**Model 3**
FR	Higher sec	1.28**	1.19	1.19	1.51***	1.25*	1.24*
		[1.07,1.54]	[0.99,1.44]	[0.98,1.44]	[1.25,1.82]	[1.02,1.53]	[1.00,1.52]
	Lower sec	1.55***	1.32*	1.33*	2.19***	1.51**	1.56***
		[1.25,1.92]	[1.04,1.66]	[1.05,1.67]	[1.72,2.78]	[1.16,1.97]	[1.20,2.04]
	Primary	1.86***	1.42**	1.46***	3.69***	2.01***	2.47***
		[1.54,2.25]	[1.15,1.77]	[1.18,1.82]	[2.76,4.95]	[1.46,2.78]	[1.78,3.43]
BCR	Higher sec	1.93**	1.89**	1.95**	1.18	1.01	1.07
		[1.23,3.03]	[1.20,2.98]	[1.23,3.08]	[0.74,1.88]	[0.62,1.66]	[0.659,1.748]
	Lower sec	2.21**	1.78*	1.94**	0.99	0.75	0.78
		[1.36,3.58]	[1.08,2.94]	[1.17,3.20]	[0.56,1.77]	[0.40,1.39]	[0.42,1.47]
	Primary	2.37***	1.59	1.96**	2.34**	1.42	1.59
		[1.54,3.66]	[0.99,2.56]	[1.21,3.15]	[1.40,3.91]	[0.78,2.58]	[0.86,2.95]
WR	Higher sec	1.06	1.03	1.04	1.53***	1.15	1.16
		[0.84,1.34]	[0.81,1.31]	[0.82,1.33]	[1.22,1.93]	[0.90,1.48]	[0.90,1.49]
	Lower sec	1.30*	1.15	1.20	1.73***	1.05	1.07
		[1.02,1.65]	[0.89,1.48]	[0.93,1.55]	[1.33,2.25]	[0.79,1.42]	[0.80,1.44]
	Primary	1.86***	1.42**	1.50**	2.70***	1.38	1.54*
		[1.48,2.32]	[1.10,1.83]	[1.16,1.94]	[1.90,3.84]	[0.94,2.03]	[1.05,2.26]

Data: census 1991, 2001 linked to national register, own calculations.

***: p < 0.001; **: p < 0.010; *: p < 0.050.

FR = Flemish Region, BCR = Brussels-Capital Region, WR = Walloon Region.

Model 1: Controlled by age/Model 2: Controlled by age and employment situation/Model 3: Controlled by age, employment situation and nationality of origin.

Some of the excess mortality in the lowest educated groups is explained by employment status. In 1991–1995, mainly the mortality rates of the primary educated were influenced by unemployment, while in 2001–2005 the all educational levels are influenced (e.g. MRR_WR-01-low sec-model1_ = 2.94 [2.42-3.58] to MRR_FR-01-low sec-model2_ = 2.27 [1.83-2.81]). After these controls, we still observe strong educational inequalities among young men, in all regions and time periods.

In model 3, we observe that in most cases, nationality of origin does not alter the results much, with the exception of the BCR in 2001–2005, showing an increase in mortality between model 2 and model 3, especially pronounced in the primary educated (MRR_BCR-01-primary-model2_ = 2.08 [1.29-3.38] - MRR_BCR-01-primary-model3_ = 2.34 [1.44-3.82]). After taking into consideration that most non-Belgians show lower mortality risks compared to Belgians [29] and generally have a lower educational attainment than Belgians, educational inequalities increase in the BCR.

Relative mortality inequalities among women also become more substantial over time (Table 6), especially in the FR. The MRR for the primary educated compared to the higher educated increased from 1.86 [1.54-2.25] in 1991–1995 to 3.69 [2.76-4.95] in 2001–2005 (model 1). Mortality rates are significantly higher for both the primary and the lower secondary educated in the FR, only significantly higher among the higher secondary educated in the WR, and even significantly lower among the lower secondary educated in the BCR in 2001–2005 (Table 2).

Few differences are statistically significant among women in the BCR in 2001–2005, which is partly due to fewer deaths compared to 1991–1995. The mortality rates are also more comparable between educational levels, except for the primary educated. These are no longer significantly different from mortality among the highest educated in model 2/3 (e.g. MRR_BCR-01-primary-model3_ = 1.59 [0.86-2.95]).

After controlling for employment in the other regions, the relative inequalities are also considerably reduced in both periods (MRR_WR-91-primary-model 2_ = 1.50 [1.16-1.94]; MRR_WR-01-primary-model 2_ = 1.54 [1.05-2.26]). Employment situation influences not only the mortality rates of the lowest educated groups, but also those of higher secondary educated women (e.g. MRR_FR-01-higher sec-model1_ = 1.51 [1.25-1.82] compared to MRR_FR-01-higher sec-model2_ = 1.24 [1.00-1.52]).

## Discussion

### Main findings & interpretation

This paper depicted educational inequalities in mortality among young adults in three Belgian regions over time. We observed a general positive trend towards substantial lower mortality in most educational groups in both men and women and found persisting inequalities over time in all regions.

Concerning regional differences in men, young-adult mortality was highest in the WR in the 1990s and 2000s. There was an overall decrease in male mortality in each observation period in the three regions, but not in each educational level. In the BCR, the spectacular decrease in mortality was partly due to a large drop in mortality both at the lower and at the higher end of the educational distribution. Over the entire observation period, mortality decreased more among the higher educated (65%) than among the primary educated (27%). In the WR we also observed stronger declines among the highest educated compared (32%) to the primary educated (8%), while there also is a small increase among the lower secondary educated (7%). In the FR we observed increases in mortality among the two lowest educated groups (16 and 21%). After controlling for nationality of origin and employment situation, the relative inequalities between regions become more comparable. Part of the male mortality decrease among the lowest educated men in the BCR can thus be attributed to the different population distribution in 2001 compared to 1991. A larger proportion of non-Belgians such as Turks and Maghrebins are living in the BCR than in the other regions. It has been well established that these groups have a lower mortality than the host population [29,30] and are overrepresented in the lower educated levels [32].

In women, young-adult mortality is a lot less common compared to men in all observed periods. For example, female mortality is 50% lower in the BCR, 53% in the FR and 57% lower in the WR compared to men in 2001–2005. Regional differences over time are similar to that of men, though the general declines over time are more modest, in part because the female mortality level in the 1990s was much lower than that of men to begin with. The only difference in general trends in all-cause mortality over time is that women in the FR still have the lowest mortality rates in 2005–2009, while the BCR showed lower mortality rates among men in this period. Concerning educational inequalities over time, we observe some sex differences in 2001–2005 in the BCR. While male mortality dropped in all educational categories, mortality among primary educated women only slightly decreased compared to the other educational groups. After accounting for employment situation, relative educational inequalities are significantly reduced, suggesting that the double deprivation of low education and not being employed makes young-adult women especially vulnerable. Earlier research also points in this direction, showing higher cause-specific mortality from most causes in primary educated women in the BCR [12].

Interpretation of persisting inequalities in mortality over time is a complex matter, as the educational composition of the population has also changed considerably over time. Both the democratization of education and the lengthening of compulsory education are at play here: there is an increased proportion of higher educated (from 23% to 33%) and a dwindling fraction of primary educated (18% to less than 5%). The primary and lower secondary educated have become a selective group of young persons. Many of these young persons have experienced or are experiencing health and/or psychological problems. This has been noticed among the total adult population in Belgium [33] as well as in different European countries [5]. It used to be common practice to sever the educational track because of job opportunities, but after the 1983 reform, which extended compulsory education until the age of 18 [34], it has become rare to quit education before finishing secondary education. Early-school leaving is then often a blurred story in which both selection and causation occur simultaneously [35,36]. A part of this group probably needs long-term or even life-long care, support and supervision in order to render them good prospects in life in general, and specifically in terms of job opportunities and health and well-being. More discussion is needed on the ways in which to give these persons the capability to enhance their state of health, despite deprivation in their young lives outside of their own choice [37].

Health selection is but one part of the story: poor school performance and problem behaviour and a deprived family background [38] are also influencing factors, leading to few or unstable job opportunities and more risk behaviour [39]. Alienation from school has been associated with health-compromising behaviour such as drug abuse and violence [40,41]. It is clear that early school leaving has severe consequences both on an individual and societal level and that preventing dropout and its negative consequences requires ensuring a close connection between educational, social and employment services both at the national and local level [38].

The question raised here is whether we can speak of increased inequalities over time, if the early school-leavers become a small and partly selective group, different from the other educational groups? As Mackenbach [42] points out, it may be possible that widening or persisting relative inequalities are the consequence of the democratization of education which made it possible for many young people to attain a certificate of higher education. Hence, the ones being left out may present a selective group in terms of health and other characteristics (see earlier paragraphs). Regarding the higher mortality rates among higher secondary educated, other mechanisms are at work. Although employment status explained some of the excess mortality in these educational groups, mortality remains 60 to 90% higher compared to the highest educated in the different regions. Multiple health advantages of each extra years of schooling, in terms of knowledge acquisition, means and social contacts are all related to these differences [16]. Although democratisation of education opened up higher education for all, there are limits to the share of the population with a higher education diploma. A broader discussion, beyond the educational system, on equal (health) opportunities for all is needed in this context.

However, all is not negative. The democratisation of education may have resulted in persisting relative inequalities, it definitely brought along absolute mortality declines. This is in line with research conducted in the general adult population [33], and shows that there is no dilution of the effect of education over time. The long-term effects of educational investments cannot be denied. As a recent article concerning increased participation in higher education concluded [43]: further improvements in educational attainment are still possible and can lead to substantial health gains.

### Regional differences in an international perspective & further research

Regional differences within Belgium have already been identified in the 1970s [21]. Thirty years later we still observe higher mortality in the WR compared to the other regions. Research not specifically focusing on mortality even found a worsening trend in health in some highly-deprived districts within the WR in the last decennia and called for community-level initiatives to bring a halt to the negative spiral this region finds itself in [44]. Our research further suggests that initiatives should be taken relatively early in life. Including meso-level organizations, such as schools, youth organisations and employment institutions, in prevention efforts would be a huge step forward in realizing long-term health benefits. We found higher mortality in the WR for each educational level compared to persons with the same educational level in the other regions in 2001–2005. Overall regional differences may partly be attributed to higher deprivation in the WR. Further research is necessary to identify other factors related to the higher mortality in the WR.

Belgium is not an isolated case in its regional mortality differences. In other European countries, considerable regional differences in absolute and relative mortality inequalities have also been observed [21]. A well-known example of within-country differences is Great Britain, with a large North–south divide between England and Scotland persisting over time [45,46]. While there is an overall decrease in mortality in Scotland, with reductions being greatest among socially advantaged groups, an increase in excess mortality has been observed among young adult men (15- to 44-year olds) in the last decades. Social patterning was found when probing into cause-specific mortality, with suicides, drug deaths, alcohol and violence as the main contributors to mortality inequalities [8,45].

In order to pinpoint domains at which future prevention efforts should be targeted in Belgium, information on cause-specific mortality is needed. Data on cause-specific mortality in the WR are however not available for the observed period. Up till now, census-linked cause-of-death information, covering the period 1991–2009, is available, available for the FR and BCR only. Unavailability of this kind of information for the WR further hampers the identification of the underlying mechanisms and, hence, the development of tailored policies and prevention campaigns. Nationwide information on cause-specific mortality will be available soon, allowing for an update of information for the WR and, hence, providing more specific insights in order to lower mortality in this region.

### Strengths & limitations

This is the first study that analysed regional mortality differences among young adults in Belgium over time, taking the three Belgian regions (FR, WR and the BCR) into account. Most studies only compare the FR and WR, leaving out an important part of Belgium. We are aware that the setting of the BCR, as a large urban area, is different from that of the other regions, especially in terms of ethnic composition of the population and other urban dynamics. Comparisons over time may not be as straightforward due to the design of our study, as the start population at each census time differs in composition because of the selective influx of migrants, especially in the BCR. To take these differences in composition into account, we controlled for nationality of origin in our regression analysis.

The high-quality data with complete follow-up on deaths and emigrations over a considerably large period make detailed analyses possible. Individually-linked mortality data furthermore rule out common numerator-denominator problems, amongst other biases [47]. Unfortunately, we do not have updated information on employment situation for the in-between periods 1995–1999 and 2005–2009. Therefore, detailed comparisons for the in-between periods are not possible.

## Conclusion

There is an overall positive trend towards lower all-cause mortality over time in Belgium. Educational inequalities are found in each observation period and region and in both men and women. The results are in line with research focusing on the persistence of social inequalities in mortality in the general adult population [4-6]. The results also show that the low educated become a small and selective group with high mortality risks. This calls for a debate on the responsibility of society in rendering support and capability to enhance the state of health and well-being of those not able to achieve a similar social status as their peers. The situation in the WR underlines the importance of good data to monitor trends in cause-specific mortality in order to develop tailored preventive efforts and and to put a halt to the worrying position the region finds itself in.

## Additional files

Additional file 1: Table S1. Absolute and relative mortality differences per 100,000 person years over time among young-adult MEN in Belgium. Additional file 2: Table S2. Absolute and relative mortality differences per 100,000 person years over time among young-adult WOMEN in Belgium.

## Abbreviations

FR: Flemish Region
BCR: Brussels-Capital Region
WR: Walloon Region
ASMR: Age-standardised mortality rate
MRR: Mortality rate ratio
SEP: Socioeconomic position
