# Supplementary material for: Educational inequalities in young-adult mortality between the 1990s and the 2000s: regional differences in Belgium
Source: Arch Public Health. 2015 Mar 16;73(1):11. doi: 10.1186/s13690-014-0059-3 (PMC4360928; doi:10.1186/s13690-014-0059-3)
Supplement: Additional file 1: Table S1. — Absolute and relative mortality differences per 100,000 person years over time among young-adult MEN in Belgium. [file 13690_2014_59_MOESM1_ESM.doc]

Additional file 1: Table S1 **Absolute and relative mortality differences per 100,000 person years over time among young-adult MEN in Belgium**

|  |  | **absolute mortality difference** | | | | **relative mortality difference** | | | |
| --- | --- | --- | --- | --- | --- | --- | --- | --- | --- |
|  |  | **1991 & 1995^a^** | **1995 & 2001^b^** | **2001 & 2005^c^** | ***Total^d^*** | **1991 & 1995^a^** | **1995 & 2001^b^** | **2001 & 2005^c^** | ***Total^d^*** |
| FR | HE | −4,1 | −15,8 | −1,8 | *−21,7* | −0,06 | −0,26 | −0,04 | *−0,34* |
|  | HSE | −3,3 | −12,9 | 7,7 | *−8,5* | −0,03 | −0,12 | 0,08 | *−0,08* |
|  | LSE | 2,9 | 8,8 | 14,6 | *26,3* | 0,02 | 0,07 | 0,11 | *0,21* |
|  | PE | −2,5 | 0,2 | 32,1 | *29,8* | −0,01 | 0,00 | 0,18 | *0,16* |
| BCR | HE | −26,0 | −29,8 | −8,8 | *−64,6* | −0,26 | −0,41 | −0,20 | *−0,65* |
|  | HSE | −23,8 | −46,9 | −12,3 | *−83,0* | −0,15 | −0,35 | −0,14 | *−0,53* |
|  | LSE | −44,7 | −38,7 | −6,7 | *−90,1* | −0,23 | −0,26 | −0,06 | *−0,46* |
|  | PE | 2,9 | −89,9 | 26,5 | *−60,5* | 0,01 | −0,40 | 0,20 | *−0,27* |
| WR | HE | −32,4 | −3,6 | 6,1 | *−29,9* | −0,35 | −0,06 | 0,11 | *−0,32* |
|  | HSE | −0,2 | −18,2 | −8,6 | *−27,0* | 0,00 | −0,13 | −0,07 | *−0,19* |
|  | LSE | −9,9 | 2,0 | 19,7 | *11,8* | −0,06 | 0,01 | 0,12 | *0,07* |
|  | PE | −0,5 | −55,5 | 33,2 | *−22,8* | 0,00 | −0,21 | 0,16 | *−0,08* |

Data: census 1991, 2001 linked to national register, own calculations.

^a^: between the period 1991–1995 and 1995–1999.

^b^: between the period 1995–1999 and 2001–2005.

^c^: between the period 2001–2005 and 2005–2009.

^d^: between the period 1991–1995 and 2005–2009.

Shaded areas = increase in mortality.

FR = Flemish Region, BCR = Brussels-Capital Region, WR = Walloon Region.

Educational level: PE= primary education; LSE= lower secondary education; HSE= higher secondary education; HE= higher education
