# Supplementary material for: Educational inequalities in young-adult mortality between the 1990s and the 2000s: regional differences in Belgium
Source: Arch Public Health. 2015 Mar 16;73(1):11. doi: 10.1186/s13690-014-0059-3 (PMC4360928; doi:10.1186/s13690-014-0059-3)
Supplement: Additional file 2: Table S2. — Absolute and relative mortality differences per 100,000 person years over time among young-adult WOMEN in Belgium. [file 13690_2014_59_MOESM2_ESM.doc]

Additional file 2: Table S2 **Absolute and relative mortality differences per 100,000 person years over time among young-adult WOMEN in Belgium**

|  |  | **absolute mortality difference between** | | | | **relative mortality difference between** | | | |
| --- | --- | --- | --- | --- | --- | --- | --- | --- | --- |
|  |  | **1991 & 1995^a^** | **1995 & 2001^b^** | **2001 & 2005^c^** | ***Total^d^*** | **1991 & 1995^a^** | **1995 & 2001^b^** | **2001 & 2005^c^** | ***Total^d^*** |
| FR | higher | 0,8 | −11,1 | −2,6 | −12,9 | 0,02 | −0,29 | −0,09 | −0,34 |
|  | higher sec | −2,3 | −4,3 | −1,0 | −7,6 | −0,05 | −0,09 | −0,02 | −0,16 |
|  | lower sec | −11,2 | 13,0 | 3,6 | 5,4 | −0,19 | 0,27 | 0,06 | 0,09 |
|  | primary | 18,3 | 15,8 | −24,4 | 9,7 | 0,26 | 0,18 | −0,23 | 0,14 |
| BCR | higher | 20,2 | −20,6 | −10,8 | −11,2 | 0,55 | −0,36 | −0,30 | −0,30 |
|  | higher sec | 10,7 | −41,1 | 15,4 | −15,0 | 0,15 | −0,49 | 0,36 | −0,21 |
|  | lower sec | −4,7 | −41,6 | 26,7 | −19,6 | −0,06 | −0,53 | 0,74 | −0,24 |
|  | primary | 3,6 | −7,3 | 22,0 | 18,3 | 0,04 | −0,08 | 0,26 | 0,20 |
| WR | higher | −4,4 | −12,9 | −6,8 | −24,1 | −0,08 | −0,26 | −0,18 | −0,44 |
|  | higher sec | −3,6 | 3,6 | −2,7 | −2,7 | −0,06 | 0,07 | −0,05 | −0,05 |
|  | lower sec | 13,2 | −18,5 | 14,5 | 9,2 | 0,19 | −0,22 | 0,22 | 0,13 |
|  | primary | 15,2 | −13,9 | 58,9 | 60,2 | 0,15 | −0,12 | 0,57 | 0,59 |

Data: census 1991, 2001 linked to national register, own calculations.

^a^: between the period 1991–1995 and 1995–1999.

^b^: between the period 1995–1999 and 2001–2005.

^c^: between the period 2001–2005 and 2005–2009.

^d^: between the period 1991–1995 and 2005–2009.

Shaded areas = increase in mortality.

FR = Flemish Region, BCR = Brussels-Capital Region, WR = Walloon Region.
